# SINR- and MI-Based Maximin Robust Waveform Design

**DOI:** 10.3390/e21010033

**Published:** 2019-01-07

**Authors:** Bin Wang, Xu Chen, Fengming Xin, Xin Song

**Affiliations:** Department of Communication Engineering, School of Computer and Communication Engineering, Northeastern University at Qinhuangdao, Qinhuangdao 066004, China

**Keywords:** cognitive radar, waveform design, signal-to-interference-plus-noise ratio (SINR), mutual information (MI)

## Abstract

Due to the uncertainties of radar target prior information in the actual scene, the waveform designed based on radar target prior information cannot meet the needs of detection and parameter estimation performance. In this paper, the optimal waveform design techniques under energy constraints for different tasks are considered. To improve the detection performance of radar systems, a novel waveform design method which can maximize the signal-to-interference-plus-noise ratio (SINR) for known and random extended targets is proposed. To improve the performance of parameter estimation, another waveform design method which can maximize the mutual information (MI) between the radar echo and the random-target spectrum response is also considered. Most of the previous waveform design researches assumed that the prior information of the target spectrum is completely known. However, in the actual scene, the real target spectrum cannot be accurately captured. To simulate this scenario, the real target spectrum was assumed to be within an uncertainty range where the upper and lower bounds are known. Then, the SINR- and MI-based maximin robust waveforms were designed, which could optimize the performance under the most unfavorable conditions. The simulation results show that the designed optimal waveforms based on these two criteria are different, which provides useful guidance for waveform energy allocation in different transmission tasks. However, under the constraint of limited energy, we also found that the performance improvement of SINR or MI in the worst case for single targets is less significant than that of multiple targets.

## 1. Introduction

As an emerging intelligent radar, cognitive radar (CR) breaks the open-loop receiving-transmitting mode of traditional radar and introduces a closed-loop system. CR can design the transmitted waveform through analyzing the information of the environment and the target, which greatly improves the detection efficiency and estimation performance of the system [1]. Electronic Warfare (EW) and Electronic Intelligence (ELINT) class systems are also widely applied in the identification process of CR [2,3,4]. In the past decades, many experts and scholars have devoted themselves to the research of transmitted waveforms to improve the detection and estimation performance of radar systems for extended targets. From the perspective of the cognitive radar system, maximizing the signal-to-interference-plus-noise ratio (SINR) can greatly improve radar detection performance for extended targets. Therefore, it is a general trend to improve the SINR through designing the transmitted waveform. For example, Cheng et al. considered maximizing the SINR through combining the transmitted waveform with the receiver filter [5], while Garren et al. proposed a transmitted waveform optimization algorithm that iteratively solves the maximum SINR [6]. The concept of entropy is derived from thermodynamics, and has been applied in fields such as antenna design [7,8]. Entropy-based mutual information (MI) is also widely used in adaptive waveform design of cognitive radars [9,10]. In 1993, the MI between a radar target and echo was first introduced by Bell to design the transmitted waveform [11], and the transmitted waveform was obtained through maximizing MI in a pure noise background. Since then, the optimal transmitted waveform design based on MI criteria has been investigated extensively. The relationship between the minimum mean square error criterion and mutual information theory is used in Guo et al.’s study [12], which optimized the transmitted waveform of cognitive radar in the context of Gaussian white noise. A cognitive radar waveform design algorithm for multiple extended targets based on mutual information is proposed in Leshem et al.’s study on the basis of a single target [13]. Radar target recognition, also known as radar signal recognition, has been widely considered [14,15,16,17], and it has been found that the target recognition ability of the radar system can be effectively improved by maximizing MI. Therefore, many waveform design methods which are committed to maximizing the MI under different conditions of the environment have been investigated [15,16,17]. In recent years, fractal-wavelets have also become a research hot spot in waveform design, and fractal-wavelet modeling and the fractal antennas theory provide useful guidance for radar waveform design [18,19,20]. However, optimal waveform designs under the environments of complex target models are not well-known, as the real target spectrum cannot be accurately captured in practice. In this paper, two novel waveform design techniques based on SINR and MI under the environment of a complex target model are presented respectively for different tasks. The maximin robust waveform design techniques which take the uncertainty of the target spectrum into account are proposed.

Our main contribution is that the imperfect estimation of target spectrum [21] is considered in designing the optimal waveform. The SINR- and MI-based maximin robust waveform design techniques are proposed respectively. To summarize, firstly, given that the real target spectrum is known, the optimal waveform design methods for the extended known target and random target based on SINR are proposed, and the optimal waveform design method for the extended random target is developed. Secondly, two novel SINR- and MI-based robust waveform design techniques are proposed respectively through considering the uncertainty of the target spectrum. In this paper, we consider both the single-target model and multiple-target model, and then the SINR- and MI-based robust waveform design techniques under the two different target models above are proposed respectively. The maximin robust waveform design methods optimize the performance of the radar transmitter under the most unfavorable conditions. In this paper, the most unfavorable condition for the different criteria is the target spectrum response which minimizes the performance of the radar system. The designed robust waveform is also analyzed in this paper. The SINR- and MI-based robust waveforms provide useful guidance for waveform energy allocation strategies. Electronic countermeasures are a variety of electronic measures and actions taken by hostile parties to weaken and destroy the use of electronic devices and to ensure the effectiveness of their own electronic devices. In this paper, the radar and target can be seen as hostile, and in order to prevent the target from being detected by the radar, the target model is blurred. Due to the uncertainties of radar target prior information, the radar transmitter design transmitted waveforms based on existing conditions make the target more detectable. Therefore, the proposed waveform design methods are effortless to realize for cognitive radar systems and applicable to complex electronic countermeasures.

## 2. Problem Formulation

In a general transmitted waveform scenario, a radar transmitter transmits a waveform, and through the reflection of the environment and the target, the receiver recovers the echo. Then, the receiver determines the echo, thereby detecting the target and estimating the target parameter.

### 2.1. Signal Model for a Known Target and Waveform Design Based on SINR

**Figure 1:**
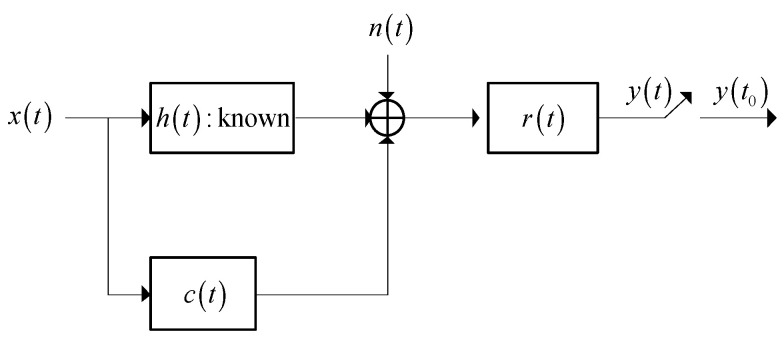
Signal model of a known target for waveform design based on the signal-to-interference-plus-noise ratio (SINR).

In this subsection, the SINR-based waveform design method which maximizes the SINR is proposed. The detection performance of a general radar system can be maximized through maximizing the SINR. As is shown in Figure 1 [22], the signal model of a known target is depicted in the radar signal processing system, where x(t) and h(t) represent the signal models of the transmitted waveform and the target respectively. X(f) represents the spectrum response of x(t), and H(f) denotes the spectrum response of h(t). r(t) represents the signal model of the receiver filter and n(t) denotes a noise signal. The mean value of n(t) is assumed to be zero and the power spectrum density (PSD) of n(t) can be denoted by $S_{nn}\left(f\right)$. Similarly, c(t) represents an interference signal, which is a Gaussian random process with the zero mean value, and the PSD of c(t) is $S_{cc}\left(f\right)$.

The total energy of the transmitted waveform is assumed to be $E_{X}$. Thus, the problem of optimizing the SINR is denoted as [22]:

(1) $$\underset{|X\left(f\right){|}^{2}}{\max}\int_{BW}\frac{{\left|H\left(f\right)X\left(f\right)\right|}^{2}}{S_{cc}\left(f\right){\left|X\left(f\right)\right|}^{2}+S_{nn}\left(f\right)}df$$

(2) $$s.t.\int_{BW}{\left|X\left(f\right)\right|}^{2}df\leq E_{X}$$

In Equation (2), BW is the bandwidth that the spectrum response of the transmitted waveform and jamming are virtually limited to. The output SINR of the matched filter in the radar receiver is used as the optimization criterion in this optimization problem [22]. The expression of SINR is expressed by the transmitted waveform, the jamming PSD, the noise PSD, and the target spectrum response.

The optimal waveform spectrum which maximizes the SINR (1) under the energy constraint (2) should satisfy [23]:

(3) $${\left|X\left(f\right)\right|}^{2}=\max\left[0,B\left(f\right)\left(A-D\left(f\right)\right)\right]$$

where:

(4) $$B\left(f\right)=\frac{\sqrt{{\left|H\left(f\right)\right|}^{2}S_{nn}\left(f\right)}}{S_{cc}\left(f\right)}$$

and:

(5) $$D\left(f\right)=\sqrt{\frac{S_{nn}\left(f\right)}{{\left|H\left(f\right)\right|}^{2}}}$$

and A denotes a constant which can be derived by the constraint of energy:

(6) $$\int {}_{BW}\max\left[0,B\left(f\right)\left(A-D\left(f\right)\right)\right]df\leq E_{X}$$

The results show that the spectrum response of the optimal waveform solution can be obtained by water injection on the function of B(f)(A−D(f)). Note that the spectra of H(f) and $S_{cc}\left(f\right)$ are supposed to be greater than zero at each sampling frequency within the bandwidth BW. Here, the water injection algorithm is based on the SINR criterion, and the energy of the designed waveform is adaptively allocated based on the energy distribution of the clutter spectrum and the target spectrum. At the sampling frequency point where the target spectrum response is strong and the clutter spectrum response is weak, more energy is allocated, but at the sampling frequency point where the target spectrum response is weak and the clutter spectrum response is strong, less energy is allocated, thereby maximizing the performance of the criterion function.

### 2.2. Signal Model for a Random Target and Waveform Design Based on SINR and MI

**Figure 2:**
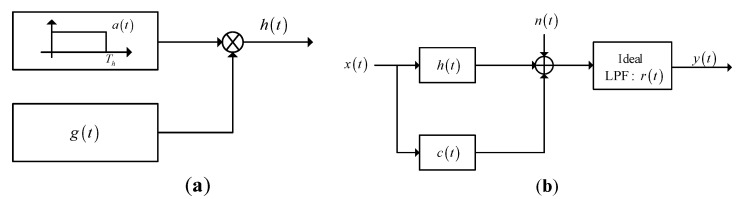
Signal model of a random target for waveform design based on SINR and mutual information (MI): (**a**) Signal model of a random target with duration $T_{h}$; (**b**) Signal model for waveform design based on SINR and MI.

The model of a random target is shown in Figure 2 [11,22], where Figure 2a illustrates that the duration of the random target is finite. In this model, g(t) denotes a generalized stationary random process and a(t) is a window function with duration $T_{h}$. Therefore, the product $\bar{h}\left(t\right)=a\left(t\right)g\left(t\right)$ is a generalized stationary random process which is supported only in the duration of [0,$T_{h}$]. The signal model of a random target is depicted in Figure 2b. The definitions of the symbols in this random-target model are the same with those in the known-target model, and the difference is that the signal model of the target model $\bar{h}\left(t\right)$ is a finite-duration random process. The energy spectrum variance (ESV) of $\bar{h}\left(t\right)$ is denoted as [11,22]:

(7) $$\sigma_{H}{}^{2}\left(f\right)=Ε\left[{\left|\bar{H}\left(f\right)-\mu_{H}\left(f\right)\right|}^{2}\right]$$

In the expression of (7), the expectation of an input entity can be denoted by E[*·*], $\bar{H}\left(f\right)$ is the spectrum response of $\bar{h}\left(t\right)$, and $\mu_{H}\left(f\right)$ denotes the mean of $\bar{H}\left(f\right)$, which is assumed to be 0.

The signal model shown in Figure 2b can be adopted in waveform design based on SINR and MI. The expression of SINR for the random extended target with limited duration can be denoted as [22]:

(8) $$SINR=\int_{-\infty }^{+\infty }\frac{\sigma_{H}{}^{2}\left(f\right){\left|X\left(f\right)\right|}^{2}}{S_{cc}\left(f\right){\left|X\left(f\right)\right|}^{2}+S_{nn}\left(f\right)}df$$

The spectrum density of SINR can be denoted as [22]:

(9) $$R_{SINR}=\frac{\sigma_{H}{}^{2}\left(f\right){\left|X\left(f\right)\right|}^{2}}{S_{cc}\left(f\right){\left|X\left(f\right)\right|}^{2}+S_{nn}\left(f\right)}$$

It is assumed that the energy of the target spectrum is mostly distributed within the range of the bandwidth BW, so the approximate expression of SINR is [22]:

(10) $$SINR=\int_{BW}\frac{\sigma_{H}{}^{2}\left(f\right){\left|X\left(f\right)\right|}^{2}}{S_{cc}\left(f\right){\left|X\left(f\right)\right|}^{2}+S_{nn}\left(f\right)}df$$

Therefore, the method of designing the transmitted waveform under the energy constraint by maximizing SINR for a random target is similar to that of a known target. The difference is that ${\left|H\left(f\right)\right|}^{2}$ in (1) is replaced by $\sigma_{H}{}^{2}\left(f\right)$ in (10). Therefore, the process and results of optimal waveform design based on SINR for the random-target model are similar to those of the known-target model and will not be described here.

Different targets have different characteristics, and each target has its specific parameters. Therefore, it is necessary to differentiate the different targets by improving the parameter estimation performance of the radar system. To improve the performance of parameter estimation, MI is adopted as the criterion to design the transmitted waveform. The expression of approximate MI based on the signal model in Figure 2b is shown as [22]:

(11) $$MI\left({\left|X\left(f\right)\right|}^{2}\right)=T_{y}\int_{BW}\ln\left[1+\frac{\sigma_{H}{}^{2}\left(f\right){\left|X\left(f\right)\right|}^{2}}{T_{y}\left(S_{cc}\left(f\right){\left|X\left(f\right)\right|}^{2}+S_{nn}\left(f\right)\right)}\right]df$$

where $T_{y}$ denotes the duration of the echo y(t). In Equation (11), the MI is expressed by the transmitted waveform, the jamming PSD, the noise PSD, and the target ESV. The designed optimal transmitted waveform should satisfy [22]:

(12) $$\underset{{\left|X\left(f\right)\right|}^{2}}{\max}\mathrm{MI}\left({\left|X\left(f\right)\right|}^{2}\right)$$

(13) $$s.t.\int_{BW}{\left|X\left(f\right)\right|}^{2}df\leq E_{X}$$

The maximization of MI means that the radar target echo contains more information about the target, which will result in rich parameter estimation performance for the radar.

The optimal waveform spectrum which maximizes the MI (12) under the energy constraint (13) should satisfy [22]:

(14) $${\left|\hat{X}\left(f\right)\right|}^{2}=\max\left[0,-R\left(f\right)+\sqrt{R^{2}\left(f\right)+S\left(f\right)\left(\hat{A}-\hat{D}\left(f\right)\right)}\right]$$

where

(15) $$\hat{D}\left(f\right)=\frac{S_{nn}\left(f\right)}{\sigma_{H}{}^{2}\left(f\right)T_{y}}$$

(16) $$R\left(f\right)=\frac{S_{nn}\left(f\right)\left(2T_{y}S_{cc}\left(f\right)+\sigma_{H}{}^{2}\left(f\right)\right)}{2S_{cc}\left(f\right)\left(T_{y}S_{cc}\left(f\right)+\sigma_{H}{}^{2}\left(f\right)\right)}$$

(17) $$S\left(f\right)=\frac{S_{nn}\left(f\right)\sigma_{H}{}^{2}\left(f\right)}{S_{cc}\left(f\right)\left(T_{y}S_{cc}\left(f\right)+\sigma_{H}{}^{2}\left(f\right)\right)}$$

and $\hat{A}$ denotes a constant which can be derived by the constraint of energy:

(18) $$\int {}_{BW}\max\left[0,-R\left(f\right)+\sqrt{R^{2}\left(f\right)+S\left(f\right)\left(\hat{A}-\hat{D}\left(f\right)\right)}\right]df\leq E_{X}$$

The results show that the spectrum response of the optimal waveform solution based on MI can be obtained by water injection, and the value of the jamming PSD is assumed to be greater than zero at each sampling frequency within the range of the bandwidth BW.

Assuming that the jamming spectrum, the noise PSD, and the target spectrum response or the target ESV are known, the optimal waveform spectrum based on SINR and MI can be designed by these prior conditions. The choice of optimal criteria is determined by the task of the radar transmitter. The designed waveform under the constraint of energy maximizes the performance of the radar system. The simulation results show that the designed transmitted waveforms based on these two criteria have different performances in waveform energy distribution within the range of the bandwidth BW.

Note that in the designed transmitted waveform methods above, the target spectrum response is assumed to be fully known, while in practice the true target spectrum is difficult to capture. When the target model is blurry, the designed waveform based on target prior information will not guarantee the performance of the radar transmitter effectively, so it is critical to minimize the loss of the performance. Therefore, robust transmitted waveform design techniques are considered next.

## 3. Maximin Robust Waveform Design

In the radar transmission environment, there may be one or multiple targets of the radar system. Therefore, a single-target scenario and a multiple-target scenario are proposed in this paper. For a single-target scenario, there is only one target to be detected in the radar transmission environment. The occurrence probability of this target is 1, and this target has a specific target spectrum. However, for a multiple-target scenario, there are multiple targets to be detected in the radar transmission environment. The occurrence probability of each target in the scenario is uncertain and the corresponding target spectrum for each target is also different, but the sum of these occurrence probabilities is 1. Then, the radar system designs the transmitted waveform based on the different target scenarios.

**Figure 3:**
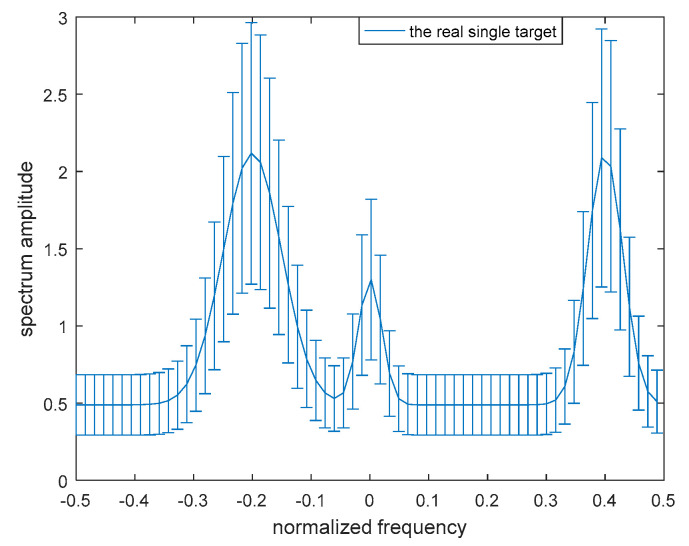
Model of the uncertainty range of the single-target spectrum.

Taking the target spectrum uncertainty into account, the band model presented in Yang and Blum’s study [23] is adopted for the single-target scenario. It is assumed that the real target spectrum exists in an uncertainty range ε, where both the upper and the lower bounds are known, that is

(19) $$H\left(f\right)\in \varepsilon =\left\{l_{k}\leq H\left(f_{k}\right)\leq u_{k},k=1,2\ldots ,K\right\}$$

where $f_{k}$ denotes the sampling frequency. The blurry model of the single target is shown in Figure 3.

**Figure 4:**
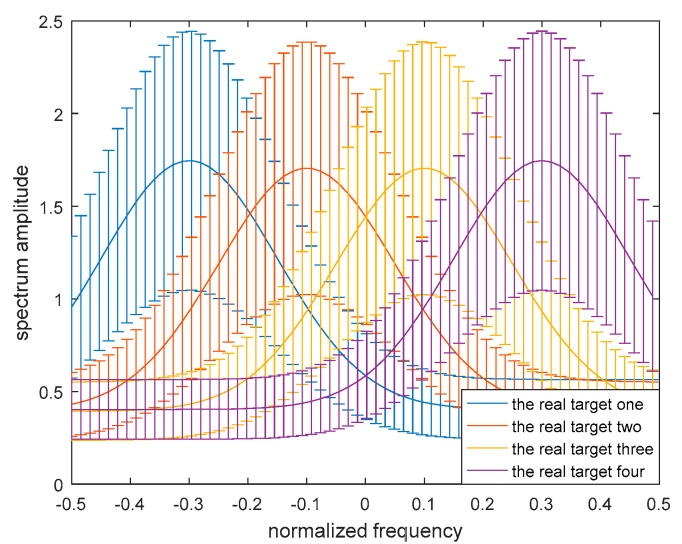
Model of the uncertainty ranges of the multiple-target spectra.

For the multiple-target scenario, each target spectrum of the multiple targets exists in an uncertainty range $\varepsilon_{i}$, where both the upper and the lower bounds are known, that is

(20) $$H_{i}\left(f\right)\in \varepsilon_{i}=\left\{l_{ik}\leq H_{i}\left(f_{k}\right)\leq u_{ik},k=1,2\ldots ,K\right\}$$

where i=1,2,3,4…, which is used to distinguish between the different targets. The uncertainty range $\varepsilon_{i}$ for each target is different. Here we assume that there are four single targets in this multiple-target scenario. The blurry target model of the multiple targets is shown in Figure 4.

In practice, the target spectrum models proposed above are widely adopted in robust waveform design because the uncertainty range can be captured through spectrum estimation [23]. The larger the difference between the upper and the lower bounds, the greater the uncertainty of the target spectrum is. Moreover, it should be noted that the differences in amplitude between the upper and the lower bounds of the blurry target spectra could be different at each sampling frequency.

For each particular target spectrum, there exists an optimal transmitted waveform. However, the real target spectrum may vary in the uncertainty range, so the maximin robust waveform design techniques based on SINR and MI are good approaches which ensure the most unfavorable performance. In this section, the maximin robust waveform design techniques based on SINR and MI are proposed respectively.

The optimization criteria of SINR or MI can be denoted by $\xi \left({\left|X\left(f\right)\right|}^{2},\sigma_{H}{}^{2}\left(f\right)\right)$. These two criteria are expressed with respect to the waveform spectrum X(f) and the target ESV $\sigma_{H}{}^{2}\left(f\right)$ or target spectrum H(f). The expressions of $\sigma_{H}{}^{2}\left(f\right)$ for a single target and for multiple targets are different, which will be given in Section 3.1.1 and Section 3.1.2 respectively. The maximin robust waveform design method should satisfy [23,24]:

(21) $$\underset{{\left|X\left(f\right)\right|}^{2}}{\max}\left\{\underset{\left|H\left(f\right)\right|\in \varepsilon }{\min}\xi ({\left|X\left(f\right)\right|}^{2},\sigma_{H}{}^{2}\left(f\right))|{}_{\int {}_{BW}{\left|X\left(f\right)\right|}^{2}df\leq E_{X}}\right\}$$

According to the theory of maximin robust signal processing [24], the solution to this maximin optimization problem is:

(22) $$\begin{array}{l} \xi \left({\left|X^{\max\min}\left(f\right)\right|}^{2},\sigma_{H}{}^{2}\left(f\right)\right)|{}_{\int {}_{BW}{\left|X^{\max\min}\left(f\right)\right|}^{2}df\leq E_{X}}\\ \geq \xi \left({\left|X^{\max\min}\left(f\right)\right|}^{2},\sigma_{H_{worst}}{}^{2}\left(f\right)\right)|{}_{\int {}_{BW}{\left|X^{\max\min}\left(f\right)\right|}^{2}df\leq E_{X}}\\ \geq \xi \left({\left|X\left(f\right)\right|}^{2},\sigma_{H_{worst}}{}^{2}\left(f\right)\right)|{}_{\int {}_{BW}{\left|X\left(f\right)\right|}^{2}df\leq E_{X}} \end{array}$$

In the above formula, for the right side of the inequality, the optimal transmitted waveform under the condition of $\sigma_{H}{}^{2}\left(f\right)=\sigma_{H_{worst}}{}^{2}\left(f\right)$ is the maximin optimal transmitted waveform. It maximizes the performance of SINR or MI at the output of the matched filter. If another transmitted waveform spectrum is used, the performance of the objective function will be degraded. The left side of the inequality means that $\sigma_{H_{worst}}{}^{2}\left(f\right)$ is the most unfavorable target ESV corresponding to the maximin optimal transmitted waveform. For all target spectra except the most unfavorable cases within the uncertainty ranges ε or $\varepsilon_{i}$, if the maximin optimal transmitted waveform spectrum ${\left|X^{\max\min}\left(f\right)\right|}^{2}$ is adopted, the performance of SINR or MI will be better than that of $\sigma_{H}{}^{2}\left(f\right)=\sigma_{H_{worst}}{}^{2}\left(f\right)$. Therefore, for the target ESV under the most unfavorable case within the uncertainty range, the maximin optimal transmitted waveform spectrum is optimal. Through limiting the performance under the most unfavorable situation, the performance loss can be effectively reduced.

### 3.1. Robust Waveform Design Based on SINR

#### 3.1.1. Robust Waveform Design Based on SINR for a Single Target

The maximin robust waveform design method based on SINR for a single target should satisfy:

(23) $$\underset{{\left|X\left(f\right)\right|}^{2}}{\max}\left\{\underset{\left|H\left(f\right)\right|\in \varepsilon }{\min}SINR({\left|X\left(f\right)\right|}^{2},\sigma_{H}{}^{2}\left(f\right))|{}_{\int {}_{BW}{\left|X\left(f\right)\right|}^{2}df\leq E_{X}}\right\}$$

**Theorem** **1.** *The maximin robust waveform for a known target which optimizes (23) can be denoted by:*

(24) $${\left|{\bar{X}}^{\max\min}\left(f\right)\right|}^{2}=\max\left[0,\bar{B}\left(f\right)\left(\bar{A}-\bar{D}\left(f\right)\right)\right]$$

where

(25) $$\bar{B}\left(f\right)=\frac{\sqrt{\sigma_{L}{}^{2}\left(f\right)S_{nn}\left(f\right)}}{S_{cc}\left(f\right)}$$

and

(26) $$\bar{D}\left(f\right)=\sqrt{\frac{S_{nn}\left(f\right)}{\sigma_{L}{}^{2}\left(f\right)}}$$

in the expression of (24). $\left|L\left(f\right)\right|=\left\{l_{k},k=1,2,\ldots ,K\right\}$ represents the lower bound of the single-target spectrum uncertainty range, where $\sigma_{L}{}^{2}\left(f\right)={\left|L\left(f\right)\right|}^{2}$ in the equations above and $\bar{A}$ is a constant which can be derived by:

(27) $$\int {}_{BW}\max\left[0,\bar{B}\left(f\right)\left(\bar{A}-\bar{D}\left(f\right)\right)\right]df\leq E_{X}$$

#### 3.1.2. Robust Waveform Design Based on SINR for Multiple Targets

The maximin robust waveform design method based on SINR for multiple targets should satisfy:

(28) $$\underset{{\left|X\left(f\right)\right|}^{2}}{\max}\left\{\underset{\left|H_{i}\left(f\right)\right|\in \varepsilon_{i}}{\min}SINR({\left|X\left(f\right)\right|}^{2},\sigma_{H}{}^{2}\left(f\right))|{}_{\int {}_{BW}{\left|X\left(f\right)\right|}^{2}df\leq E_{X}}\right\}$$

**Theorem** **2.** *The maximin robust waveform for a known target which optimizes (28) can be denoted by:*

(29) $${\left|{\overset{⌢}{X}}^{\max\min}\left(f\right)\right|}^{2}=\max\left[0,\overset{⌢}{B}\left(f\right)\left(\overset{⌢}{A}-\overset{⌢}{D}\left(f\right)\right)\right]$$

where

(30) $$\overset{⌢}{B}\left(f\right)=\frac{\sqrt{\sigma_{L}{}^{2}\left(f\right)S_{nn}\left(f\right)}}{S_{cc}\left(f\right)}$$

and

(31) $$\overset{⌢}{D}\left(f\right)=\sqrt{\frac{S_{nn}\left(f\right)}{\sigma_{L}{}^{2}\left(f\right)}}$$

in the expression of (29). $\left|L_{i}\left(f\right)\right|=\left\{l_{ik},k=1,2,\ldots ,K\right\}$ represents the lower bound of the ith target spectrum uncertainty range, where $\sigma_{L}{}^{2}\left(f\right)=\sum_{i=1}^{M}P_{i}{\left|L_{i}\left(f\right)\right|}^{2}-{\left|\sum_{i=1}^{M}P_{i}L_{i}\left(f\right)\right|}^{2}$ [22] in the Equations (29)–(31), M denotes the number of targets, $P_{i}$ denotes the occurrence probability of the ith target, and $\overset{⌢}{A}$ is a constant which can be derived by:

(32) $$\int {}_{BW}\max\left[0,\overset{⌢}{B}\left(f\right)\left(\overset{⌢}{A}-\overset{⌢}{D}\left(f\right)\right)\right]df\leq E_{X}$$

Note that the optimization problem in (28), which maximizes SINR for the model of multiple targets through designing the transmitted waveform under the energy constraint, is similar to the optimization problem in (23). The difference is that the expression of $\sigma_{H}{}^{2}\left(f\right)$ varies from $\sigma_{H}{}^{2}\left(f\right)={\left|H\left(f\right)\right|}^{2}$ to $\sigma_{H}{}^{2}\left(f\right)=\sum_{i=1}^{M}P_{i}{\left|H_{i}\left(f\right)\right|}^{2}-{\left|\sum_{i=1}^{M}P_{i}H_{i}\left(f\right)\right|}^{2}$.

The proof of Theorems 1 and 2 is as follows:

In order to prove the conclusion above, the optimal problem should satisfy:

(33) $$\begin{array}{l} SINR\left({\left|X^{\max\min}\left(f\right)\right|}^{2},\sigma_{H}{}^{2}\left(f\right)\right)|{}_{\int {}_{BW}{\left|X^{\max\min}\left(f\right)\right|}^{2}df\leq E_{X}}\\ \geq SINR\left({\left|X^{\max\min}\left(f\right)\right|}^{2},\sigma_{H_{worst}}{}^{2}\left(f\right)\right)|{}_{\int {}_{BW}{\left|X^{\max\min}\left(f\right)\right|}^{2}df\leq E_{X}}\\ \geq SINR\left({\left|X\left(f\right)\right|}^{2},\sigma_{H_{worst}}{}^{2}\left(f\right)\right)|{}_{\int {}_{BW}{\left|X^{\max\min}\left(f\right)\right|}^{2}df\leq E_{X}} \end{array}$$

Therefore, the right side of the inequality in (33) will be proved firstly as follows. To review, the expression of SINR can be denoted by:

(34) $$SINR\left({\left|X\left(f\right)\right|}^{2}\right)=\int_{BW}\frac{\sigma_{H}{}^{2}\left(f\right){\left|X\left(f\right)\right|}^{2}}{S_{cc}\left(f\right){\left|X\left(f\right)\right|}^{2}+S_{nn}\left(f\right)}df$$

The expression of $\sigma_{H}{}^{2}\left(f\right)$ in (34) is different for a single target or for multiple targets. It is assumed that the most unfavorable target spectrum |L(f)| can be captured. Therefore, the most unfavorable target ESV $\sigma_{L}{}^{2}\left(f\right)$ is available. Similarly, $\sigma_{L}{}^{2}\left(f\right)$ is the lower bound of $\sigma_{H}{}^{2}\left(f\right)$. This optimization problem is equivalent to assuming that the real target spectrum |L(f)| is known, then the transmitted waveform can be designed by maximizing the SINR.

The Lagrangian multiplier method is a method that can find the extreme value of a function under constraints. The main idea is to associate the constraint function with the original function by introducing a new parameter λ (i.e., Lagrange multiplier), so that it can be formulated into equations with the same number of variables, and then the solution of each variable that obtains the extreme value of the original function can be found.

Now we adopt the Lagrangian multiplier method to determine the objective function:

(35) $$L\left({\left|X\left(f\right)\right|}^{2},\lambda \right)=\int_{BW}\frac{\sigma_{L}{}^{2}\left(f\right){\left|X\left(f\right)\right|}^{2}}{S_{cc}\left(f\right){\left|X\left(f\right)\right|}^{2}+S_{nn}\left(f\right)}df+\lambda \left[E_{X}-\int_{BW}{\left|X\left(f\right)\right|}^{2}df\right]$$

This is equivalent to maximizing $L\left({\left|X\left(f\right)\right|}^{2}\right)$ by solving ${\left|X\left(f\right)\right|}^{2}$, and thus Equation (35) can be converted into:

(36) $$L\left({\left|X\left(f\right)\right|}^{2},\lambda \right)=\int_{BW}\frac{\sigma_{L}{}^{2}\left(f\right){\left|X\left(f\right)\right|}^{2}}{S_{cc}\left(f\right){\left|X\left(f\right)\right|}^{2}+S_{nn}\left(f\right)}df-\lambda \int_{BW}{\left|X\left(f\right)\right|}^{2}df$$

In Equation (36), $L\left({\left|X\left(f\right)\right|}^{2}\right)$ can be denoted by:

(37) $$L\left({\left|X\left(f\right)\right|}^{2}\right)=\frac{\sigma_{L}{}^{2}\left(f\right){\left|X\left(f\right)\right|}^{2}}{S_{cc}\left(f\right){\left|X\left(f\right)\right|}^{2}+S_{nn}\left(f\right)}df-\lambda {\left|X\left(f\right)\right|}^{2}$$

Next, we derive $L\left({\left|X\left(f\right)\right|}^{2}\right)$ to ${\left|X\left(f\right)\right|}^{2}$:

(38) $$\frac{dL\left({\left|X\left(f\right)\right|}^{2}\right)}{d{\left|X\left(f\right)\right|}^{2}}=\frac{\sigma_{L}{}^{2}\left(f\right)S_{nn}\left(f\right)}{{\left(S_{cc}\left(f\right){\left|X\left(f\right)\right|}^{2}+S_{nn}\left(f\right)\right)}^{2}}df-\lambda$$

Setting $\frac{dL\left({\left|X\left(f\right)\right|}^{2}\right)}{d{\left|X\left(f\right)\right|}^{2}}$ to zero yields the ${\left|X\left(f\right)\right|}^{2}$ value which maximizes (34), where ${\left|X\left(f\right)\right|}^{2}$ is given by:

(39) $${\left|X\left(f\right)\right|}^{2}=-\frac{S_{nn}\left(f\right)}{S_{cc}\left(f\right)}\pm \sqrt{\frac{\sigma_{L}{}^{2}\left(f\right)S_{nn}\left(f\right)}{\lambda {\left|S_{cc}\left(f\right)\right|}^{2}}}$$

We let $\bar{A}=\sqrt{\frac{1}{\lambda }}$ to ensure that ${\left|X\left(f\right)\right|}^{2}$ is positive. Then, ${\left|X\left(f\right)\right|}^{2}$ can be expressed by:

(40) $${\left|X^{\max\min}\left(f\right)\right|}^{2}=\max\left[0,\frac{\sqrt{\sigma_{L}{}^{2}\left(f\right)S_{nn}\left(f\right)}}{S_{cc}\left(f\right)}\left(A-\sqrt{\frac{S_{nn}\left(f\right)}{\sigma_{L}{}^{2}\left(f\right)}}\right)\right]$$

Equation (40) can also be written as:

(41) $${\left|X^{\max\min}\left(f\right)\right|}^{2}=\max\left[0,\bar{B}\left(f\right)\left(\bar{A}-\bar{D}\left(f\right)\right)\right]$$

where

(42) $$\bar{B}\left(f\right)=\frac{\sqrt{\sigma_{L}{}^{2}\left(f\right)S_{nn}\left(f\right)}}{S_{cc}\left(f\right)}$$

and

(43) $$\bar{D}\left(f\right)=\sqrt{\frac{S_{nn}\left(f\right)}{\sigma_{L}{}^{2}\left(f\right)}}$$

in the expression of (41). Therefore, we obtain:

(44) $$\begin{array}{l} SINR\left({\left|X^{\max\min}\left(f\right)\right|}^{2},\sigma_{H_{worst}}{}^{2}\left(f\right)\right)|{}_{\int {}_{BW}{\left|X^{\max\min}\left(f\right)\right|}^{2}df\leq E_{X}}\\ \geq SINR\left({\left|X\left(f\right)\right|}^{2},\sigma_{H_{worst}}{}^{2}\left(f\right)\right)|{}_{\int {}_{BW}{\left|X^{\max\min}\left(f\right)\right|}^{2}df\leq E_{X}} \end{array}$$

Then, we can prove that $H_{worst}\left(f\right)=\left|L\left(f\right)\right|$ is the most unfavorable target spectrum and that $\sigma_{Hworst}{}^{2}\left(f\right)=\sigma_{L}{}^{2}\left(f\right)$ is the most unfavorable target ESV. By substituting the designed waveform spectrum result into the SINR expression of (34) for any H(f)∈ε or $H_{i}\left(f\right)\in \varepsilon_{i}$, the integral is approximated by summation, which is:

(45) $$\begin{array}{l} SINR\left({\left|X^{\max\min}\left(f\right)\right|}^{2},\sigma_{H}{}^{2}\left(f\right)\right)\\ =\sum_{k=1}^{K}\Delta f\frac{\sigma_{H}{}^{2}\left(f_{k}\right)\cdot \max\left[0,\frac{\sqrt{\sigma_{L}{}^{2}\left(f_{k}\right)S_{nn}\left(f_{k}\right)}}{S_{cc}\left(f_{k}\right)}\left(\bar{A}-\sqrt{\frac{S_{nn}\left(f_{k}\right)}{\sigma_{L}{}^{2}\left(f_{k}\right)}}\right)\right]}{S_{cc}\left(f\right)\cdot \max\left[0,\frac{\sqrt{\sigma_{L}{}^{2}\left(f_{k}\right)S_{nn}\left(f_{k}\right)}}{S_{cc}\left(f_{k}\right)}\left(\bar{A}-\sqrt{\frac{S_{nn}\left(f_{k}\right)}{\sigma_{L}{}^{2}\left(f_{k}\right)}}\right)\right]+S_{nn}\left(f_{k}\right)}\\ =\sum_{k=1}^{K}\Delta f\frac{\sigma_{H}{}^{2}\left(f_{k}\right)\cdot \max\left[0,\frac{\sqrt{\sigma_{L}{}^{2}\left(f_{k}\right)S_{nn}\left(f_{k}\right)}}{S_{cc}\left(f_{k}\right)}\left(\bar{A}-\sqrt{\frac{S_{nn}\left(f_{k}\right)}{\sigma_{L}{}^{2}\left(f_{k}\right)}}\right)\right]}{\max\left[S_{nn}\left(f_{k}\right),\bar{A}\cdot \sqrt{\sigma_{L}{}^{2}\left(f_{k}\right)S_{nn}\left(f_{k}\right)}\right]}\\ \geq \sum_{k=1}^{K}\Delta f\frac{\sigma_{L}{}^{2}\left(f_{k}\right)\cdot \max\left[0,\frac{\sqrt{\sigma_{L}{}^{2}\left(f_{k}\right)S_{nn}\left(f_{k}\right)}}{S_{cc}\left(f_{k}\right)}\left(\bar{A}-\sqrt{\frac{S_{nn}\left(f_{k}\right)}{\sigma_{L}{}^{2}\left(f_{k}\right)}}\right)\right]}{\max\left[S_{nn}\left(f_{k}\right),\bar{A}\cdot \sqrt{\sigma_{L}{}^{2}\left(f_{k}\right)S_{nn}\left(f_{k}\right)}\right]}\\ =SINR\left({\left|X^{\max\min}\left(f\right)\right|}^{2},\sigma_{H_{worst}}{}^{2}\left(f\right)\right) \end{array}$$

where Δf denotes the interval of the sampling frequency. Thus, $H_{worst}\left(f\right)=\left|L\left(f\right)\right|$ is the most unfavorable target spectrum which minimizes the SINR, and similarly the most unfavorable target ESV is $\sigma_{Hworst}{}^{2}\left(f\right)=\sigma_{L}{}^{2}\left(f\right)$, which completes the proof.

### 3.2. Robust Waveform Design Based on MI

#### 3.2.1. Robust Waveform Design Based on MI for a Single Target

The maximin robust waveform design method based on MI for single target should satisfy:

(46) $$\underset{{\left|\tilde{X}\left(f\right)\right|}^{2}}{\max}\left\{\underset{\left|H\left(f\right)\right|\in \varepsilon }{\min}MI({\left|\tilde{X}\left(f\right)\right|}^{2},\sigma_{H}{}^{2}\left(f\right))|{}_{\int {}_{BW}{\left|\tilde{X}\left(f\right)\right|}^{2}df\leq E_{X}}\right\}$$

**Theorem** **3.** *The maximin robust waveform for a known target which optimizes (46) can be denoted by:*

(47) $${\left|{\tilde{X}}^{\max\min}\left(f\right)\right|}^{2}=\max\left[0,-\tilde{R}\left(f\right)+\sqrt{{\tilde{R}}^{2}\left(f\right)+\tilde{S}\left(f\right)\left(\tilde{A}-\tilde{D}\left(f\right)\right)}\right]$$

where

(48) $$\tilde{R}\left(f\right)=\frac{S_{nn}\left(f\right)\left(2T_{y}S_{cc}\left(f\right)+\sigma_{L}{}^{2}\left(f\right)\right)}{2S_{cc}\left(f\right)\left(T_{y}S_{cc}\left(f\right)+\sigma_{L}{}^{2}\left(f\right)\right)}$$

(49) $$\tilde{D}\left(f\right)=\frac{S_{nn}\left(f\right)}{\sigma_{L}{}^{2}\left(f\right)T_{y}}$$

and

(50) $$\tilde{S}\left(f\right)=\frac{S_{nn}\left(f\right)\sigma_{L}{}^{2}\left(f\right)}{S_{cc}\left(f\right)\left(T_{y}S_{cc}\left(f\right)+\sigma_{L}{}^{2}\left(f\right)\right)}$$

where $\sigma_{L}{}^{2}\left(f\right)={\left|L\left(f\right)\right|}^{2}$ in the equations above, which is the same as $\sigma_{L}{}^{2}\left(f\right)$ in Section 3.1.1, and $\tilde{A}$ is a constant which can be derived by:

(51) $$\int {}_{BW}\max\left[0,-\tilde{R}\left(f\right)+\sqrt{{\tilde{R}}^{2}\left(f\right)+\tilde{S}\left(f\right)\left(\tilde{A}-\tilde{D}\left(f\right)\right)}\right]df\leq E_{X}$$

#### 3.2.2. Robust Waveform Design Based on MI for Multiple Targets

The maximin robust waveform design method based on MI for multiple targets should satisfy:

(52) $$\underset{{\left|\tilde{X}\left(f\right)\right|}^{2}}{\max}\left\{\underset{\left|H_{i}\left(f\right)\right|\in \varepsilon_{i}}{\min}MI({\left|\tilde{X}\left(f\right)\right|}^{2},\sigma_{H}{}^{2}\left(f\right))|{}_{\int {}_{BW}{\left|\tilde{X}\left(f\right)\right|}^{2}df\leq E_{X}}\right\}$$

**Theorem** **4.** *The maximin robust waveform for a known target which optimizes (52) can be denoted by:*

(53) $${\left|{\overset{⌣}{X}}^{\max\min}\left(f\right)\right|}^{2}=\max\left[0,\overset{⌣}{R}\left(f\right)+\sqrt{{\overset{⌣}{R}}^{2}\left(f\right)+\overset{⌣}{S}\left(f\right)\left(\overset{⌣}{A}-\overset{⌣}{D}\left(f\right)\right)}\right]$$

where

(54) $$\overset{⌣}{R}\left(f\right)=\frac{S_{nn}\left(f\right)\left(2T_{y}S_{cc}\left(f\right)+\sigma_{L}{}^{2}\left(f\right)\right)}{2S_{cc}\left(f\right)\left(T_{y}S_{cc}\left(f\right)+\sigma_{L}{}^{2}\left(f\right)\right)}$$

(55) $$\overset{⌣}{D}\left(f\right)=\frac{S_{nn}\left(f\right)}{\sigma_{L}{}^{2}\left(f\right)T_{y}}$$

and

(56) $$\overset{⌣}{S}\left(f\right)=\frac{S_{nn}\left(f\right)\sigma_{L}{}^{2}\left(f\right)}{S_{cc}\left(f\right)\left(T_{y}S_{cc}\left(f\right)+\sigma_{L}{}^{2}\left(f\right)\right)}$$

in the expression of (53). $\sigma_{L}{}^{2}\left(f\right)=\sum_{i=1}^{M}P_{i}{\left|L_{i}\left(f\right)\right|}^{2}-{\left|\sum_{i=1}^{M}P_{i}L_{i}\left(f\right)\right|}^{2}$ in Equations (54)–(56), which is the same as $\sigma_{L}{}^{2}\left(f\right)$ in Section 3.1.2, and $\overset{⌣}{A}$ is a constant which can be derived by:

(57) $$\int {}_{BW}\max\left[0,\overset{⌣}{R}\left(f\right)+\sqrt{{\overset{⌣}{R}}^{2}\left(f\right)+\overset{⌣}{S}\left(f\right)\left(\overset{⌣}{A}-\overset{⌣}{D}\left(f\right)\right)}\right]df\leq E_{X}$$

The proof of Theorems 3 and 4 is as follows:

In order to prove the conclusion above, the optimal problem should satisfy:

(58) $$\begin{array}{l} MI\left({\left|{\tilde{X}}^{\max\min}\left(f\right)\right|}^{2},\sigma_{H}{}^{2}\left(f\right)\right)|{}_{\int {}_{BW}{\left|{\tilde{X}}^{\max\min}\left(f\right)\right|}^{2}df\leq E_{X}}\\ \geq MI\left({\left|{\tilde{X}}^{\max\min}\left(f\right)\right|}^{2},\sigma_{H_{worst}}{}^{2}\left(f\right)\right)|{}_{\int {}_{BW}{\left|{\tilde{X}}^{\max\min}\left(f\right)\right|}^{2}df\leq E_{X}}\\ \geq MI\left({\left|\tilde{X}\left(f\right)\right|}^{2},\sigma_{H_{worst}}{}^{2}\left(f\right)\right)|{}_{\int {}_{BW}{\left|{\tilde{X}}^{\max\min}\left(f\right)\right|}^{2}df\leq E_{X}} \end{array}$$

Therefore, the right side of the inequality in (58) will be proved firstly as follows. To review, the expression of MI can be denoted by:

(59) $$MI\left({\left|\tilde{X}\left(f\right)\right|}^{2}\right)=T_{y}\int_{BW}\ln\left[1+\frac{\sigma_{H}{}^{2}\left(f\right){\left|\tilde{X}\left(f\right)\right|}^{2}}{T_{y}\left(S_{cc}\left(f\right){\left|\tilde{X}\left(f\right)\right|}^{2}+S_{nn}\left(f\right)\right)}\right]df$$

The expression of $\sigma_{H}{}^{2}\left(f\right)$ in (59) is still different for a single target or for multiple targets. It is assumed that the most unfavorable target spectrum can be captured and the most unfavorable target ESV is available, which is the same as the proof of Theorems 1 and 2. This optimization problem is equivalent to assuming that the real target spectrum |L(f)| is known, and that the transmitted waveform can be designed by maximizing the MI.

We adopt the Lagrangian multiplier method to determine the objective function:

(60) $$L\left({\left|\tilde{X}\left(f\right)\right|}^{2},\lambda \right)=T_{y}\int_{BW}\ln\left[1+\frac{\sigma_{L}{}^{2}\left(f\right){\left|\tilde{X}\left(f\right)\right|}^{2}}{T_{y}\left(S_{cc}\left(f\right){\left|\tilde{X}\left(f\right)\right|}^{2}+S_{nn}\left(f\right)\right)}\right]df+\lambda \left[E_{X}-\int_{BW}{\left|\tilde{X}\left(f\right)\right|}^{2}df\right]$$

This is equivalent to maximizing $L\left({\left|\tilde{X}\left(f\right)\right|}^{2}\right)$ by solving ${\left|\tilde{X}\left(f\right)\right|}^{2}$. Therefore, Equation (60) can be converted into:

(61) $$L\left({\left|\tilde{X}\left(f\right)\right|}^{2},\lambda \right)=T_{y}\int_{BW}\ln\left[1+\frac{\sigma_{L}{}^{2}\left(f\right){\left|\tilde{X}\left(f\right)\right|}^{2}}{T_{y}\left(S_{cc}\left(f\right){\left|\tilde{X}\left(f\right)\right|}^{2}+S_{nn}\left(f\right)\right)}\right]df-\lambda \int_{BW}{\left|\tilde{X}\left(f\right)\right|}^{2}df$$

In Equation (61), $L\left({\left|\tilde{X}\left(f\right)\right|}^{2}\right)$ can be denoted by:

(62) $$L\left({\left|\tilde{X}\left(f\right)\right|}^{2}\right)=T_{y}\cdot \ln\left[1+\frac{\sigma_{L}{}^{2}\left(f\right){\left|\tilde{X}\left(f\right)\right|}^{2}}{T_{y}\left(S_{cc}\left(f\right){\left|\tilde{X}\left(f\right)\right|}^{2}+S_{nn}\left(f\right)\right)}\right]df-\lambda {\left|\tilde{X}\left(f\right)\right|}^{2}$$

Deriving $L\left({\left|\tilde{X}\left(f\right)\right|}^{2}\right)$ to ${\left|\tilde{X}\left(f\right)\right|}^{2}$ and setting the derivative function to zero yields:

(63) $$\lambda =\frac{S_{nn}\left(f\right)\sigma_{L}{}^{2}\left(f\right)}{A\left(f\right){\left|\tilde{X}\left(f\right)\right|}^{4}+E\left(f\right){\left|\tilde{X}\left(f\right)\right|}^{2}+C\left(f\right)}$$

where

(64) $$A\left(f\right)=\frac{S_{cc}\left(f\right)\cdot \left(T_{y}S_{cc}\left(f\right)+\sigma_{L}{}^{2}\left(f\right)\right)}{T_{y}}$$

(65) $$E\left(f\right)=\frac{S_{nn}\left(f\right)\cdot \left(2T_{y}S_{cc}\left(f\right)+\sigma_{L}{}^{2}\left(f\right)\right)}{T_{y}}$$

and

(66) $$C\left(f\right)={\left|S_{nn}\left(f\right)\right|}^{2}$$

in the expression of (63). By setting $\tilde{A}=\frac{T_{y}}{\lambda }$ to ensure that ${\left|\tilde{X}\left(f\right)\right|}^{2}$ is positive, ${\left|\tilde{X}\left(f\right)\right|}^{2}$ can be expressed by:

(67) $${\left|{\tilde{X}}^{\max\min}\left(f\right)\right|}^{2}=\max\left[0,-\tilde{R}\left(f\right)+\sqrt{{\tilde{R}}^{2}\left(f\right)+\tilde{S}\left(f\right)\left(\tilde{A}-\tilde{D}\left(f\right)\right)}\right]$$

The waveform spectrum result in (67) is the result after first-order Taylor approximation,

where:

(68) $$\tilde{R}\left(f\right)=\frac{S_{nn}\left(f\right)\left(2T_{y}S_{cc}\left(f\right)+\sigma_{L}{}^{2}\left(f\right)\right)}{2S_{cc}\left(f\right)\left(T_{y}S_{cc}\left(f\right)+\sigma_{L}{}^{2}\left(f\right)\right)}$$

(69) $$\tilde{D}\left(f\right)=\frac{S_{nn}\left(f\right)}{\sigma_{L}{}^{2}\left(f\right)T_{y}}$$

and

(70) $$\tilde{S}\left(f\right)=\frac{S_{nn}\left(f\right)\sigma_{L}{}^{2}\left(f\right)}{S_{cc}\left(f\right)\left(T_{y}S_{cc}\left(f\right)+\sigma_{L}{}^{2}\left(f\right)\right)}$$

in the expression of (67).

Therefore, we obtain:

(71) $$\begin{array}{l} MI\left({\left|{\tilde{X}}^{\max\min}\left(f\right)\right|}^{2},\sigma_{H_{worst}}{}^{2}\left(f\right)\right)|{}_{\int {}_{BW}{\left|{\tilde{X}}^{\max\min}\left(f\right)\right|}^{2}df\leq E_{X}}\\ \geq MI\left({\left|\tilde{X}\left(f\right)\right|}^{2},\sigma_{H_{worst}}{}^{2}\left(f\right)\right)|{}_{\int {}_{BW}{\left|{\tilde{X}}^{\max\min}\left(f\right)\right|}^{2}df\leq E_{X}} \end{array}$$

Then, we prove that $H_{worst}\left(f\right)=\left|L\left(f\right)\right|$ is the most unfavorable target spectrum and $\sigma_{Hworst}{}^{2}\left(f\right)=\sigma_{L}{}^{2}\left(f\right)$ is the most unfavorable target ESV. By substituting the designed waveform spectrum result into the MI expression of (59) for any H(f)∈ε or $H_{i}\left(f\right)\in \varepsilon_{i}$, the integral is approximated by summation, which is:

(72) $$\begin{array}{l} MI\left({\left|{\tilde{X}}^{\max\min}\left(f\right)\right|}^{2},\sigma_{H}{}^{2}\left(f\right)\right)\\ =T_{y}\cdot \sum_{k=1}^{K}\Delta f\cdot \ln\left[1+\frac{\sigma_{H}{}^{2}\left(f_{k}\right){\left|{\tilde{X}}^{\max\min}\left(f_{k}\right)\right|}^{2}}{T_{y}\left(S_{cc}\left(f_{k}\right){\left|{\tilde{X}}^{\max\min}\left(f_{k}\right)\right|}^{2}+S_{nn}\left(f_{k}\right)\right)}\right]\\ =T_{y}\cdot \sum_{k=1}^{K}\Delta f\cdot \ln\left[1+\frac{\sigma_{H}{}^{2}\left(f_{k}\right)\cdot \max\left(0,G\left(f_{k}\right)\right)}{T_{y}\cdot \max\left(S_{nn}\left(f_{k}\right),S_{cc}\left(f_{k}\right)G\left(f_{k}\right)+S_{nn}\left(f_{k}\right)\right)}\right]\\ \geq T_{y}\cdot \sum_{k=1}^{K}\Delta f\cdot \ln\left[1+\frac{\sigma_{L}{}^{2}\left(f_{k}\right)\cdot \max\left(0,G\left(f_{k}\right)\right)}{T_{y}\cdot \max\left(S_{nn}\left(f_{k}\right),S_{cc}\left(f_{k}\right)G\left(f_{k}\right)+S_{nn}\left(f_{k}\right)\right)}\right]\\ =MI\left({\left|{\tilde{X}}^{\max\min}\left(f\right)\right|}^{2},\sigma_{H_{worst}}{}^{2}\left(f\right)\right) \end{array}$$

In the expression of (72), we define that $G\left(f_{k}\right)=-\tilde{R}\left(f_{k}\right)+\sqrt{{\tilde{R}}^{2}\left(f_{k}\right)+\tilde{S}\left(f_{k}\right)\left(\tilde{A}-\tilde{D}\left(f_{k}\right)\right)}$. Thus, $H_{worst}\left(f\right)=\left|L\left(f\right)\right|$ is the most unfavorable target spectrum which minimizes the MI, and similarly the most unfavorable target ESV is $\sigma_{Hworst}{}^{2}\left(f\right)=\sigma_{L}{}^{2}\left(f\right)$. This completes the proof.

Therefore, the most unfavorable target spectrum which minimizes the MI is $H_{worst}\left(f\right)=\left|L\left(f\right)\right|$, and similarly the most unfavorable target ESV is $\sigma_{Hworst}{}^{2}\left(f\right)=\sigma_{L}{}^{2}\left(f\right)$. This solution guarantees that:

(73) $$\begin{array}{l} MI\left({\left|{\tilde{X}}^{\max\min}\left(f\right)\right|}^{2},\sigma_{H}{}^{2}\left(f\right)\right)|{}_{\int {}_{BW}{\left|{\tilde{X}}^{\max\min}\left(f\right)\right|}^{2}df\leq E_{X}}\\ \geq MI\left({\left|{\tilde{X}}^{\max\min}\left(f\right)\right|}^{2},\sigma_{H_{worst}}{}^{2}\left(f\right)\right)|{}_{\int {}_{BW}{\left|{\tilde{X}}^{\max\min}\left(f\right)\right|}^{2}df\leq E_{X}} \end{array}$$

which denotes the left side of (58). Therefore, Theorems 3 and 4 are proved.

In the designed robust waveform based on SINR and MI, the most unfavorable target spectrum is the lower bound of the target uncertainty range. Therefore, the optimal waveform design considering the upper and lower bounds of the uncertainty range can greatly improve the performance of the radar system. Here, we only need to consider the lower bound of the uncertainty range.

The optimal transmitted waveform design techniques based on SINR and MI provide useful guidance for waveform energy allocation in different radar tasks. In maximin robust waveform design, we care more about how the designed transmitted waveform based on SINR and MI is affected by the uncertainty of the target spectrum. According to the previous researches, we can conclude that if the target model is blurred, that is, the real target spectrum exists in an uncertainty range, the transmitted waveforms designed based on these two criteria will show the same behavior. In the uncertainty range, the smaller the amplitude of the target spectrum, the worse the performance of SINR and MI, which will reduce the performance of the radar system. The maximin robust method can guarantee the performance under the most unfavorable case, so the robust optimal transmitted waveform can be designed based on the lower bound of the uncertainty range of the target spectrum.

## 4. Simulation and Results

**Figure 5:**
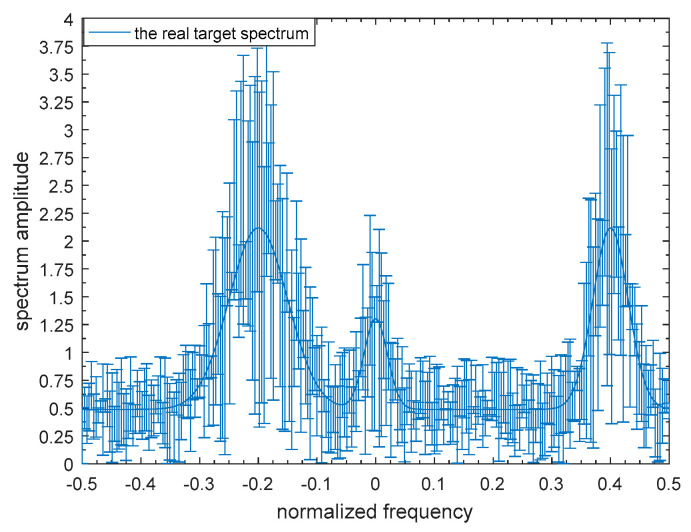
Bounded single-target spectrum samples.

**Figure 6:**
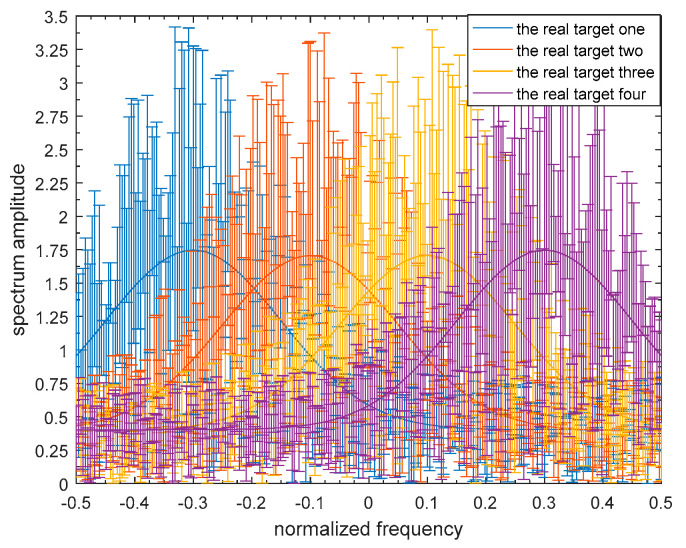
Bounded multiple-target spectrum samples.

To demonstrate the validity of the robust transmitted waveform techniques based on SINR and MI for a single target and for multiple targets as proposed above, a lot of simulation analyses were performed in this paper. The uncertainty ranges of the single-target and multiple-target spectra are presented in Figure 5 and Figure 6 respectively. The real single-target and multiple-target spectra are denoted by the solid lines, and the performance of the maximin robust waveform based on SINR and MI will be displayed later. The main energy of the real single-target spectrum is allocated near the normalized frequencies −0.2, 0, and 0.4. For each target of the nominal multiple targets, the main energy is allocated near the normalized frequencies −0.3, −0.1, 0.1, and 0.3, and the corresponding occurrence probability of each target is 0.1, 0.2, 0.3, and 0.4 respectively. The upper and the lower bounds at each sampling frequency are denoted by the deviation bounds. The amplitude of the upper bound is the real amplitude with a random value added, and similarly the lower bound is the real amplitude with a random value subtracted. The random value corresponding to the upper bound obeys a uniform distribution between zero and one at each sampling frequency.

**Figure 7:**
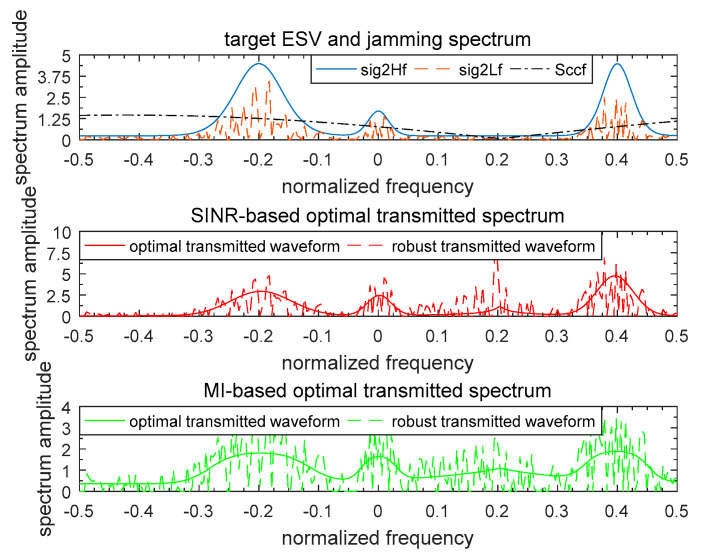
Waveform spectrum results of a single target.

**Figure 8:**
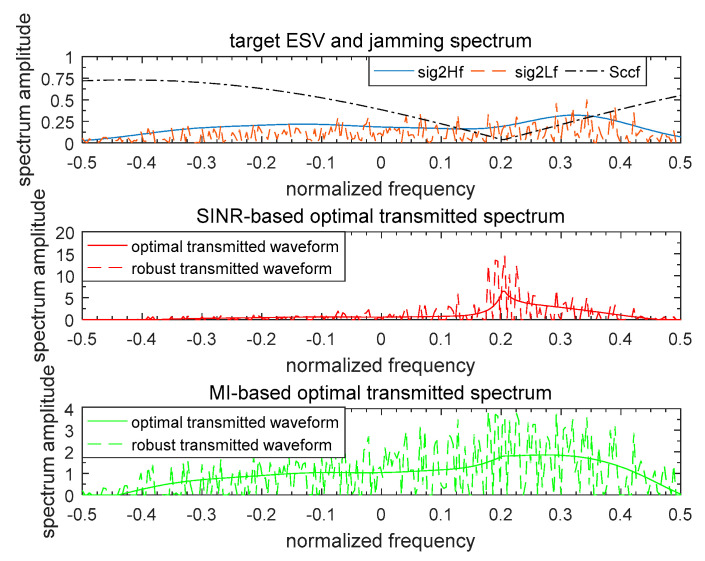
Waveform spectrum results of multiple targets.

Figure 7 and Figure 8 illustrate the waveform spectrum results based on SINR and MI. The top panels of Figure 7 and Figure 8 show the real target ESV, the most unfavorable target ESV, and the spectrum responses of signal-dependent jamming of a single target and multiple targets respectively. For the model of the random target, the spectrum response of the random target was considered to be the same as the known target here. It was assumed that the real random target ESV was $\sigma_{H}{}^{2}\left(f\right)={\left|H\left(f\right)\right|}^{2}$, and so the most unfavorable target ESV was accordingly $\sigma_{L}{}^{2}\left(f\right)={\left|L\left(f\right)\right|}^{2}$. Therefore, the two designed optimal waveform spectra based on SINR and MI for the known target were the same as the random target. The total energy of the waveform spectrum was 1 W. In Figure 7 and Figure 8, the optimal waveform spectra and robust waveform spectra based on SINR and MI are presented in the middle panel and bottom panel respectively. As it is expected, for both a single target or multiple targets, the optimal waveform based on SINR places its main energy into few frequency bands, whereas the optimal waveform based on MI places its energy into multiple frequency bands.

**Figure 9:**
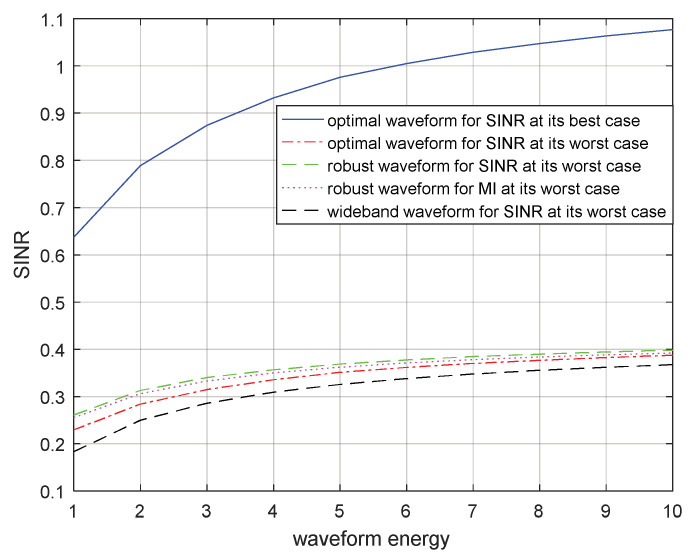
SINR performance for a robust waveform of a single target.

**Figure 10:**
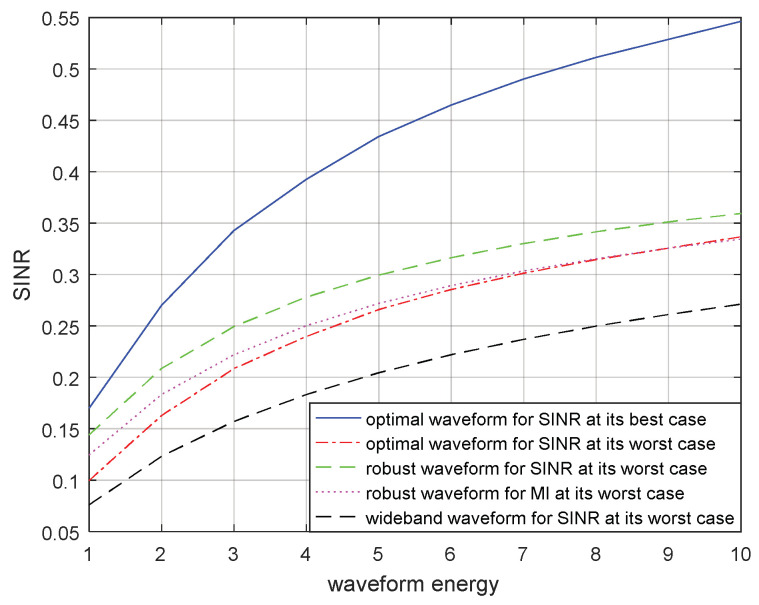
SINR performance for a robust waveform of multiple targets.

Suppose that the energy constraint of the transmitted waveform increases from 1 to 10 W. In Figure 9 and Figure 10, the SINRs corresponding to the optimal transmitted waveform based on SINR for the real target spectrum, the optimal transmitted waveform based on SINR for the most unfavorable target spectrum, the robust transmitted waveform based on SINR in the most unfavorable case, the robust waveform based on MI in the most unfavorable case, and the wide-band transmitted waveform under the most unfavorable case are compared for a single target and for multiple targets respectively. The SINR obtained by using the optimal transmitted waveform for the real target spectrum was optimal because the real target spectrum was assumed to be known, which could be used to design the corresponding optimal transmitted waveform. Therefore, it had the largest SINR and would achieve the best detection performance of the radar. When the target spectrum was most unfavorable within the uncertainty range, that is, the lower bound of the range, the optimal transmitted waveform corresponding to the real target spectrum could also be used to obtain the SINR for the most unfavorable target spectrum or the most unfavorable target ESV which is shown in Figure 9 and Figure 10 respectively. As we had estimated, the SINR corresponding to the robust transmitted waveform under the most unfavorable case was between the two SINRs mentioned above. That was because there was relatively little prior information of the target spectrum for the robust transmitted waveform. However, it worked better than the SINR corresponding to the optimal transmitted waveform for the most unfavorable target spectrum, because the most unfavorable performance was improved by using maximin robust techniques. Subsequently, the SINRs corresponding to the robust transmitted waveform based on MI for the most unfavorable target spectrum are shown in Figure 9 and Figure 10 respectively. Due to the different criteria, the performance of the resulting SINRs were poor compared to those of the SINRs corresponding to the robust transmitted waveform based on SINR. The SINRs corresponding to the wide-band transmitted waveform under the most unfavorable case are also presented in Figure 9 and Figure 10, where the wide-band waveforms indicate that the transmitted waveform spectrum was a straight line over the entire frequency band and did not contain the information about the target, noise, and signal-dependent interference.

**Figure 11:**
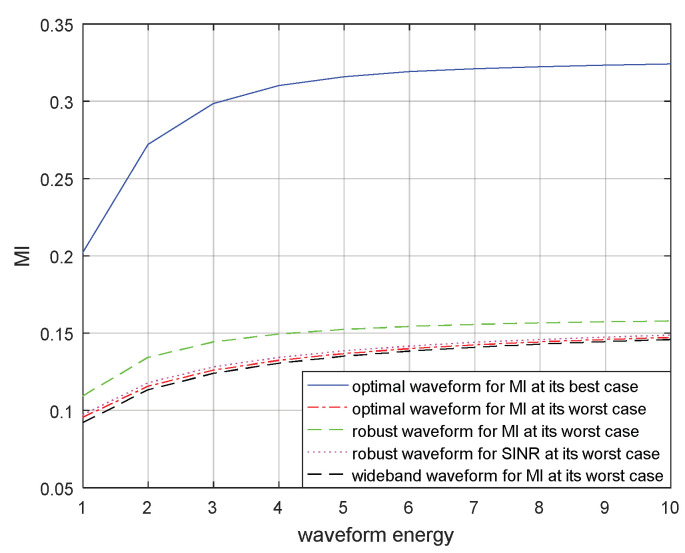
MI performance for the robust waveform of a single target.

**Figure 12:**
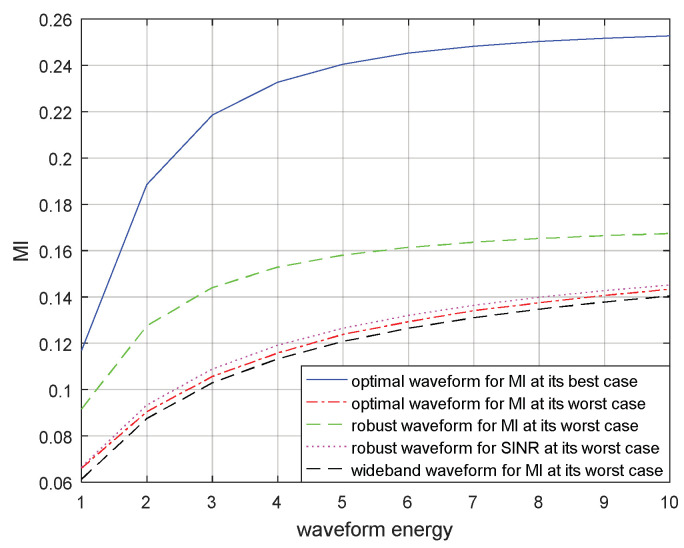
MI performance for the robust waveform of multiple targets.

Similarly, the MIs corresponding to the optimal transmitted waveform based on MI for the real target spectrum, the optimal transmitted waveform based on MI for the most unfavorable target spectrum, the robust transmitted waveform based on MI in the most unfavorable case, the robust waveform based on SINR in the most unfavorable case, and the wide-band transmitted waveform under the most unfavorable case are compared for a single target and for multiple targets in Figure 11 and Figure 12 respectively. The results demonstrate that the optimal transmitted waveform based on MI for the real target spectrum had the biggest MI. The lack of prior information of the blurred target model resulted that the wide-band transmitted waveform under the most unfavorable case having the smallest MI. The MI corresponding to the robust transmitted waveform based on SINR under the most unfavorable case was the third biggest MI, due to different criteria. The performance of the MI corresponding to the robust transmitted waveform under the most unfavorable case was better than the MI corresponding to the optimal transmitted waveform under the most unfavorable case, because the most unfavorable performance of MI was maximized. However, it was worse than that of the MI corresponding to the optimal transmitted waveform for the real target spectrum, because the model of target spectrum is uncertain.

The optimal and robust transmitted waveform spectra provide useful guidance for waveform energy allocation. The figures above show that the designed transmitted waveforms based on these two criteria have different performances in waveform energy distribution. The waveform based on SINR places its main energy into few frequency bands, whereas the waveform based on MI places its energy into multiple frequency bands. In addition, the robust waveform design techniques based on SINR and MI above can improve the performance of the radar system under the most unfavorable case. If the real target spectrum is within the target uncertainty range, the performances of SINR and MI will be better than that of the robust waveform under the most unfavorable case, or at least the same as the most unfavorable case. However, when adopting other waveform spectra, the performances of SINR and MI are worse than that of adopting the robust waveform. From the results in Figure 9 and Figure 10 or Figure 11 and Figure 12, we should note that the performance improvements of SINR and MI under the most unfavorable case for a single target are less significant than those of multiple targets. One reason for this is that the uncertainty of the multiple targets is larger than that of the single target.

## 5. Conclusions

The optimal waveform design techniques based on SINR for known- and random-target models, and MI for random-target models were proposed in this paper, which assumed that the real target spectrum is known. These waveform design techniques based on SINR and MI are suitable for the environment of limited energy. Then, the uncertainty ranges of the single-target and multiple-target spectra were considered. The real target spectrum was assumed to be within an uncertainty range where the upper and lower bounds are known. Then, the maximin robust waveform based on SINR and MI were designed according to the model of a blurred target. The results demonstrate that the maximin robust waveform design based on SINR and MI proposed in this paper can improve the performance of the radar system efficiently and provide useful guidance for energy distribution. We also found that the performance improvement of SINR or MI under the most unfavorable case for a single target is less significant than that of multiple targets.
